# Chemical synthesis of left arm of *Chlamydomonas reinhardtii* mitochondrial genome and *in vivo* functional analysis

**DOI:** 10.3389/fmicb.2022.1064497

**Published:** 2022-12-22

**Authors:** Quan Wang, Haolin Luo, Jieyi Zhuang, Xinyi Li, Danqiong Huang, Zhangli Hu, Guiying Zhang

**Affiliations:** ^1^Guangdong Technology Research Center for Marine Algal Bioengineering, College of Life Sciences and Oceanography, Shenzhen University, Shenzhen, China; ^2^Key Laboratory of Optoelectronic Devices and Systems of Ministry of Education and Guangdong Province, College of Optoelectronic Engineering, Shenzhen University, Shenzhen, China; ^3^Shenzhen Engineering Laboratory for Marine Algal Biotechnology, Longhua Innovation Institute for Biotechnology, Shenzhen University, Shenzhen, China; ^4^Southern Marine Science and Engineering Guangdong Laboratory (Guangzhou), Guangzhou, China

**Keywords:** *Chlamydomonas reinhardtii*, mitochondrial genome, synthetic biology, assemble, left arm, functionalization, heteroplasmic, homoplasmic

## Abstract

*Chlamydomonas reinhardtii* is a photosynthetic eukaryote showing great industrial potential. The synthesis and *in vivo* function of the artificial *C. reinhardtii* genome not only promotes the development of synthetic biology technology but also supports industries that utilize this algae. Mitochondrial genome (MtG) is the smallest and simplest genome of *C. reinhardtii* that suits synthetic exploration. In this article, we designed and assembled a synthetic mitochondria left arm (syn-LA) genome sharing >92% similarity to the original mitochondria genome (OMtG) left arm, transferred it into the respiratory defect strain *cc-2654*, screened syn-LA containing transformants from recovered dark-growth defects using PCR amplification, verified internal function of syn-LA *via* western blot, detected heteroplasmic ratio of syn-LA, tried promoting syn-LA into homoplasmic status with paromomycin stress, and discussed the main limitations and potential solutions for this area of research. This research supports the functionalization of a synthetic mitochondrial genome in living cells. Although further research is needed, this article nevertheless provides valuable guidance for the synthesis of eukaryotic organelle genomes and opens possible directions for future research.

## Introduction

Synthetic biology is a newly emerging discipline that combines biology, engineering, and chemistry (Lisa, 2020; Guha et al., 2022). The main research aims of synthetic biology is to redesign and reconstruct biological pathways, natural macromolecules, and regulating systems using engineering and bottom-up strategies. The ultimate goal is to construct predictable “modified cells” or “artificial life” with beneficial biological functions (Garner, 2021; Darvishi, 2022). Genomic DNA, the main carrier of genetic information and the instruction center for all life activities, is often regarded as the starting point of “life reconstruction”. Genome synthesis has been accomplished in viruses and prokaryotes including poliovirus, φX174 bacteriophage, T7 bacteriophage, severe acute respiratory syndrome coronavirus (SARS-CoV), *Mycoplasma genitalium*, West Nile Virus, and severe acute respiratory syndrome coronavirus 2 (SARS-CoV-2). (Cello et al., 2002; Smith et al., 2003; Chan et al., 2005; Becker et al., 2008; Gibson et al., 2008; Orlinger et al., 2010; Tran et al., 2020). In addition, the first synthetic artificial cell was successfully synthesized in 2010 and minimized the genome size from 1.08 M (JCVI-syn1.0) to 531 kb (JCVI-syn3.0) in 2016 (Gibson et al., 2010a; Hutchison et al., 2016). In the same year, the *Escherichia coli* genome was recoded (Ostrov et al., 2016). In recent years, research on the synthesis of the *Saccharomyces cerevisiae* genome has progressed, with more details of genome structure, function, and evolution providing knowledge for biotechnological applications (Dymond et al., 2011; Annaluru et al., 2014; Richardson et al., 2017; Romano et al., 2019; Darvishi, 2022). However, the development of mitochondrial genome synthesis has been relative slow. So far, only three synthetic mitochondria have been reported, in mouse and *Phaeodactylum tricornutum*. However, the research conducted mitochondrial genome synthesis and assembly without *in vivo* function testing (Itaya et al., 2008; Gibson et al., 2010b; Cochrane et al., 2020).

Considering the unique characteristics of mitochondria, including double-wrapped membranes, unique internal structure, numerous copies, and ultra-small size (0.5–1 μm), challenges are obvious for this specific organelle’s transformation (Bernt et al., 2013; Wu et al., 2020). It must be considered that for a long time, mitochondrial transformation was almost impossible, especially in higher plant cells (Li et al., 2021). Multi-layer membranes greatly hinder the penetration of DNA molecules (Ackerman et al., 1992; Andrews et al., 1999; Sloan et al., 2012; Haddad, 2021). Despite more than 30 years of research on mitochondrial transformation, poor transformation efficiency in sporadic species with the potential of mitochondrial transformation is something researchers are still investigating (Johnston et al., 1988; Remacle et al., 2006; Verdin et al., 2010). In addition to the obstacles from multi-membranes, ultra-small dimensions are another challenge for mitochondrial transformation. Further, to transform the heteroplasmic genome into homoplasmic status is another technical barrier. Integration of foreign genes into the original genome inevitably leads to the coexistence of wild- and transformed-type DNA in mitochondria (Mühleip et al., 2021). However, how to end the coexistence status is still unknown. Beyond the abovementioned technical limitations, other techniques, such as effective screening approaches, quick sorting strategies, and accurate detection, are yet to be established. Therefore, exploring and developing mitochondrial transformation technologies are important, enriching eukaryotic genome synthetic research and providing a basis for repairing mitochondrial diseases.

The model microalga *C. reinhardtii* is a photosynthetic unicellular eukaryote with characteristics that differ from yeast, and it has been widely exploited by the pharmaceutical, nutraceutical, cosmetic, food, feed, chemical, and diesel industries. *Chlamydomonas reinhardtii* possesses a mitochondrial genome that shows the possibility of being transformed. The 15.8 kb *C. reinhardtii* MtG encodes only eight functional proteins, making it appropriate for eukaryotic genome synthesis research (Vahrenholz et al., 1993). In addition, the linear architecture, succinct DNA information with few redundant and repetitive sequences, and suitable GC content (45%) further facilitate its genome synthesis (Yang et al., 1985; Gray, 2012; Chandel, 2021). In addition, comprehensive mitochondrial sequencing information, advanced commercial oligonucleotide synthesis techniques, and abundant restriction enzymes enable the synthesis and assembly of artificial mitochondrial genomes.

In this study, we designed and assembled an artificial syn-LA of the *C. reinhardtii* mitochondrial genome and successfully transformed it into respiratory defective algal cells. The heteroplasmic ratio of syn-LA in transformants was detected, and *in vivo* function was confirmed by western blotting. In addition, attempts to end heteroplasmic status using antibiotic stress were attempted. The upgraded foreign DNA delivery system based on carbon nanotube, mitochondrial transcription activator-like effector nucleases (mito-TALEN)-based system of ending heteroplasmic status, and intelligent sorting system based on microscopic difference are discussed. As this is the first study of mitochondrial genome synthesis and functionalization in living cells, this study will be valuable for providing guidance on the synthesis of eukaryotic organelle genomes.

## Results and discussion

### Design, synthesis, and assembly of the left arm of the mitochondrial genome

**Figure 1:**
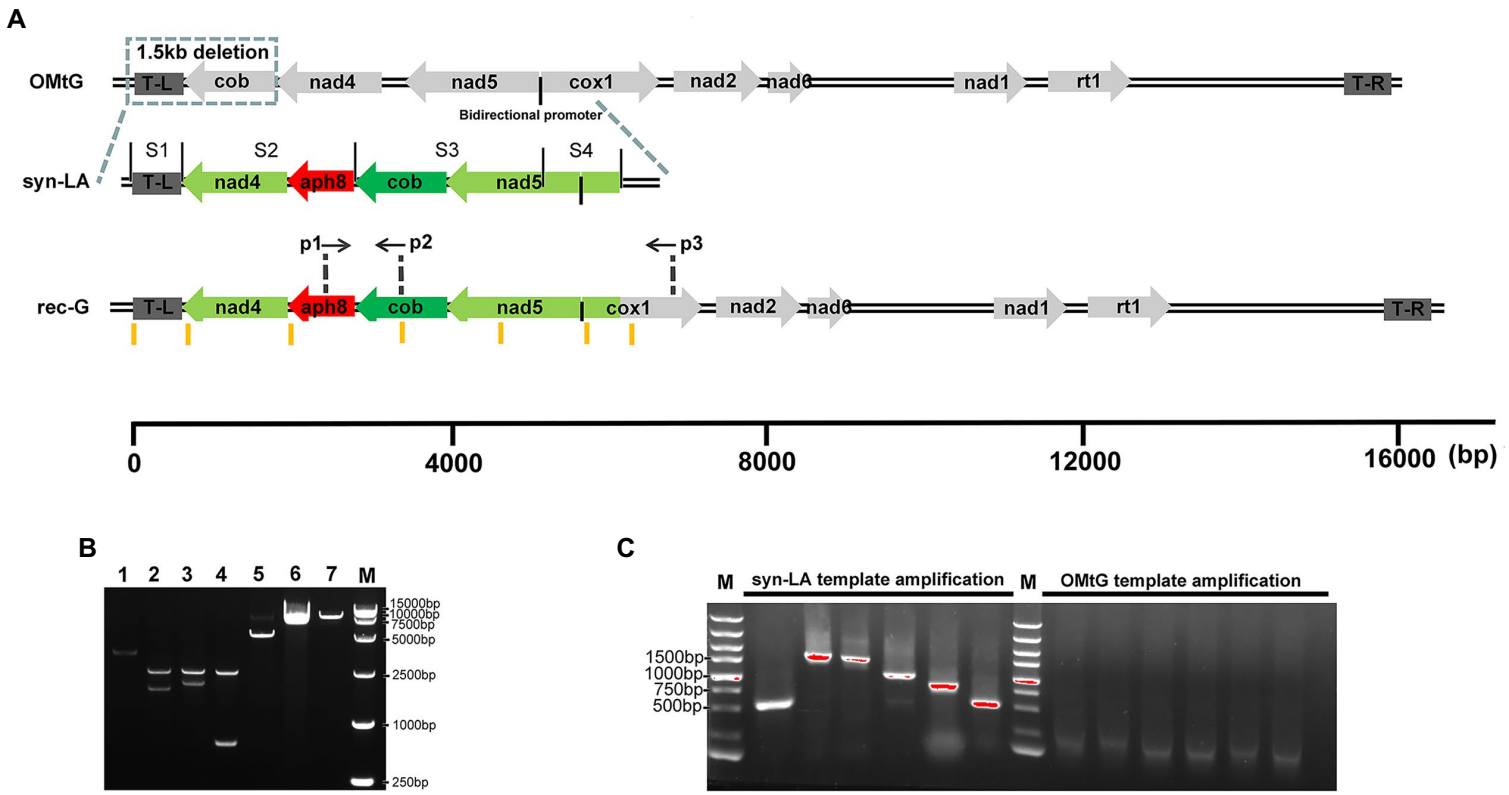
Design, synthesis, and assembly of the left arm of the mitochondrial genome. **(A)** Schematic diagram of the original mitochondrial genome (OMtG), the synthetic left arm (syn-LA), and the recombinant genome (rec-G). The box on OMtG represents a ~ 1.5 kb deletion in the *cc-2654* mutant; the black line between *nad5* and *cox1* indicates a bidirectional promoter; S1–S4 on syn-LA indicate synthesized segments; p1, p2, and p3 on rec-G indicate the primers used to identify transformants; the brown lines below rec-G represent six pairs of primers (Amp1-Amp12) used to distinguish OMtG and syn-LA (Supplementary Table 1). **(B)** Agarose gel electrophoresis of assembly syn-LA, lane 1, 2, 3, 4, 5, 6, and 7 indicate *pPstI-S1-NotI-XhoI-SgrDI-SacI* digest with NotI and XhoI (3,488 + 7 bp), *pNotI-S2-XhoI* digest with NotI and XhoI (2,924 + 2,180 bp), *pXhoI-S3-SgrDI* digest with XhoI and SgrDI (2,924 + 2,633 bp), *pSgrDI-S4-SacI* digest with SgrDI and *SacI* (2,924 + 723 bp), *pPstI-S1-NotI-S2-XhoI-SgrDI-SacI* digest with XhoI and SgrDI (5,661 + 7 bp), *pPstI-S1-NotI-S2-XhoI-S3-SgrDI-SacI* digest with SgrDI and *SacI* (8,283 + 11 bp), and *pPstI-S1-NotI-S2-XhoI-S3-SgrDI-S4-SacI* digest with *SacI* (9,006 bp) respectively. **(C)** Agarose gel electrophoresis of PCR amplification result or wild-type gDNA and pGEMT-LA plasmids with Amp1-Amp12 primers.

To ensure functional activity and facilitate future research, syn-LA was designed with maximum similarity to the original *C. reinhardtii* mitochondria genome (NC_001638.1). Detailed labels and modification for identification and future updates were introduced (Figure 1A; Table 1). Specifically, stop codons were replaced; the TAA codon of *cob*, *nad4*, and *nad5* were altered to TGA. A further 19 synonymous substitutions referring to nine amino acids, comprising Thr, Val, Ser, Ala, Phe, Arg, His, Leu, and Cys, were equally introduced. Considering that the original cob gene is located at the left end, we shifted it to the intergenic region of *nad4* and *nad5*, as there are two orthologous arms available in contrast with a single arm at the left end. In addition, aiming to assist with transformant screening and achieve homoplasmic status of syn-LA, an 804 bp paromomycin resistance gene *aph8* was inserted to the 1,928–2,731 site of syn-LA. Furthermore, to facilitate assembly and convenient future upgrades, four unique restriction sites (PstI, NotI, XhoI, and PmeI) were inserted into the 1–6, 551–558, 2,732–2,737, and 3,890–3,897 sites, respectively. Finally, a 6,090 bp syn-LA sequence sharing 92% identity with the OMtG left arm was obtained.

**Table 1:** Modifications on syn-LA.

**Substitutions**	**Sequence/Alter**	**Site (1-6,090 bp)**	**Description**
1	C → T	581	TAA → TGA
2	G → A	1,037	Synonymous substitution of Thr
3	A → G	1,040	Synonymous substitution of Val
4	AGA → GCT	1,046–1,048	Synonymous substitution of Ser
5	CT → GA	1,536–1,537	Synonymous substitution of Ser
6	A → G	1,538	Synonymous substitution of Ala
7	T → C	2,751	TAA → TGA
8	G → A	3,387	Synonymous substitution of Ser
9	G → A	3,390	Synonymous substitution of Phe
10	T → C	3,908	TAA → TGA
11	A → G	3,911	Synonymous substitution of Ala
12	A → G	3,914	Synonymous substitution of Arg
13	G → A	4,577	Synonymous substitution of His
14	A → G	4,580	Synonymous substitution of Thr
15	A → T	4,586	Synonymous substitution of Leu
16	G → A	4,589	Synonymous substitution of His
17	GGA → ACT	5,102–5,104	Synonymous substitution of Ser
18	A → G	5,105	Synonymous substitution of Cys
19	A → T	5,108	Synonymous substitution of Val
20	T → C	5,650	Synonymous substitution of Thr
21	TC → AG	5,651–5,652	Synonymous substitution of Ser
22	T → C	5,656	Synonymous substitution of His
**Insertions**	**/**	**/**	**/**
1	CTGCAG	1–6	PstI site
2	GCGGCCGC	551–558	NotI site
3	CTCGAGCCGGAATTCCGGCT	2,732–2,737	XhoI，EcoRI site and protect base
4	GTTTAAAC	3,890–3,897	PmeI site
5	aph8 ORF	1928–2,731	Resistance label
**Shifting**	**/**	**/**	**/**
1	*cob ORF*	2,751–3,896	Shift from 545–1,690 on OMtG to 2,751–3,896 on syn-LA
**Deletion**	**/**	**/**	**/**
1	263 bp intergenic region	3,051-3,313(OMtG)	Intergenic region between nad4 and nad5 on OMtG

Benefiting from technological advances, nucleotide molecule synthesis is now extremely convenient, and numerous biotechnology companies provide precise and high-quality DNA synthesis services. The syn-LA sequence was synthesized in four segments (S1–S4) and cloned into the pGEM-T vector by General Biol Company. Specifically, *pPstI-S1-NotI-XhoI-SgrDI-SacI*, *pNotI-S2-XhoI*, *pXhoI-S3-SgrDI*, and *pSgrDI-S4-SacI* four elementary plasmids were obtained. S2, S3, and S4 fragments were then sequentially cut off from the opposite plasmid through double digests and added to *pPstI-S1-NotI-XhoI-SgrDI-SacI* in turn (Figure 1B). Thus, final *pPstI-S1-NotI-S2-XhoI-S3-SgrDI-S4-SacI* containing full-length syn-LA was obtained. To further confirm the assembled syn-LA genomes, PCR amplification was performed (Figure 1C). As primers were designed at positions with insertions or substitutions, the OMtG template could not be bound or extended, making it distinguishable from the synthetic left arm (syn-LA; Supplementary Table 1).

### Mitochondrial transformation, transformants screening, and function validation

**Figure 2:**
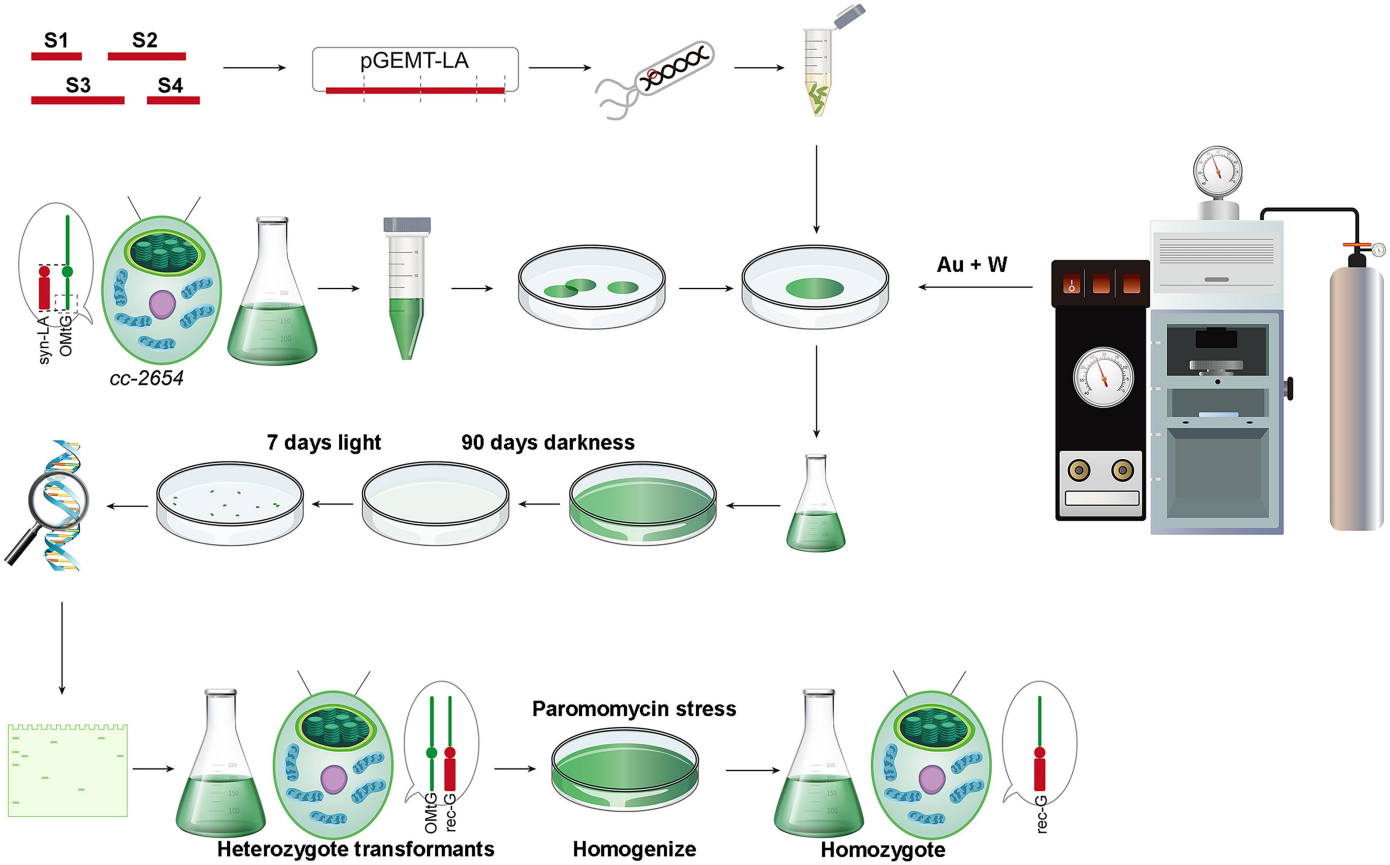
Workflow of the transformation of syn-LA into mitochondria; using paromomycin increases syn-LA ratio in transformants.

Mitochondria mutant strain *cc-2654*, which does not grow on acetate plates in darkness, was selected as the recipient strain (Randolph-Anderson et al., 1993; Vahrenholz et al., 1993). The lack of ability of *cc-2654* can be restored by complementing the intact mitochondrial *cob* gene and hence facilitating transformant screening. In consideration of the low efficiency of the existing biolistic mitochondria transformation method, we adopted a modified method. Microcarriers used in mainstream biolistic equipment were restricted to the micron level. The smallest commodity microcarrier is a particle of 0.6 μm gold (Au), which larger than mitochondria (0.5–1 μm). Such particles often cause severe physical damage of the mitochondria when bombarded. Irreversible mitochondrial damage greatly reduces the possibility of successful transfection (Kleele et al., 2021). Thus, a mixture of 0.6 μm gold and 50 nm tungsten (W) powder were applied as our DNA microcarrier to reduce physical damage and prevent aggregation (Figure 2).

**Figure 3:**
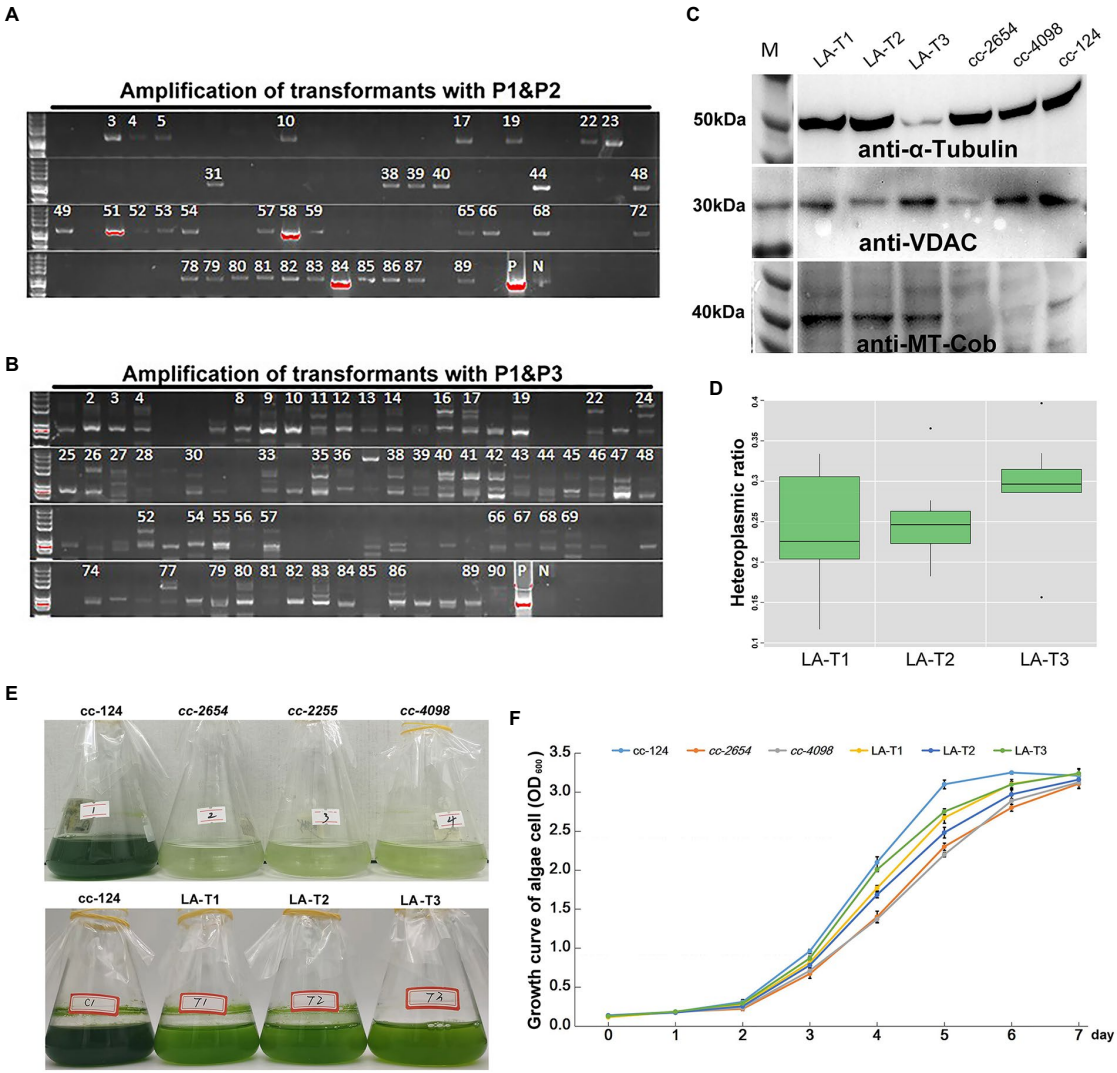
Screening and identification of syn-LA transformants. **(A)** Agarose gel electrophoresis of p1&p2 amplification results. **(B)** Agarose gel electrophoresis of p1&p3 amplification results. pGEMT-LA plasmid DNA and *cc-124* gDNA were included as positive (P) and negative (N) controls, respectively. Strains marked with numbers are candidate positive transformants. **(C)** Western blotting results for LA-T1, LA-T2, LA-T3, *cc-2654*, *cc-4098*, and *cc-124* mitochondrial protein with anti-α-tubulin, anti-VDAC, and anti-MT-cob antibodies. **(D)** HR (Heteroplasmic ratio) of syn-LA in LA-T1, LA-T2, and LA-T3 strains. **(E)** Phenotype of 15 days in darkness of cc-124, *cc-2654*, *cc-2255*, *cc-4098*, LA-T1, LA-T2, and LA-T3. **(F)** Growth curve statistics of cc-124, *cc-2654*, *cc-4098*, LA-T1, LA-T2, and LA-T3; bars represent average values of three replicates ± standard deviation (SD).

Using the modified method, we successfully obtained hundreds of candidate algal colonies after 90 days of dark screening (Figure 2 and Supplementary Figures 1 A,B). To further confirm the presence of syn-LA in those colonies, PCR amplification with two pairs of primers, p1&p2 and p1&p3, was conducted. Primers p1 and p2 are located on *aph8* and *cob* of syn-LA, while primer p3 is located outside syn-LA on *cox1* ORF. PCR fragments generated from p1&p2 were as expected, i.e., identical to positive controls. Hence, the results indicated that syn-LA was successfully delivered into *cc-2654* cells. The expected 4,499 bp fragment generated from primer pair p1&p3 suggested the success replacement of syn-LA at the corresponding region of OMtG (Figures 3A,B). In addition, some unexpected PCR products were also observed from amplification results of p1&p3, noticed possibly because both the syn-LA and OMtG genome exist in the cell.

To further confirm whether syn-LA was indeed functionalized inside algal cells, mitochondrial proteins were extracted from *cc-2654*, *cc-4098* (allelic mutant of *cc-2654*), wild-type cc-124 strains, and three syn-LA transformants (LA-T1, LA-T2, and LA-T3) to execute western blotting. The quality of extracted protein was evidenced by the presence of expected 50 and 32 kDa proteins for all six samples after immunoblotting with anti-α-tubulin and anti-VDAC/porin antibodies (Figure 3C). Meanwhile, the 43 kDa protein encoded by *cob* gene, which should be missing in the *cc-2654* and *cc-4098* sample, was detected in LA-T1, LA-T2, and LA-T3. Therefore, *cob* in syn-LA was successfully expressed, indicating that syn-LA should be functionalized *in vivo*.

As there are usually hundreds or even thousands of mitochondria DNA molecules present in each cell, an occasional mutation or transgenic event is unlikely to alter all mitochondria DNA copies. Thus, the mitochondrial genome can present in a heteroplasmic state within a single cell (Machado et al., 2015; Filograna et al., 2019). Considering the excess bands detected by p1&p3, we also wanted to evaluate the ratio of syn-LA genome in transformants based on quantitative real-time PCR. The single copy nuclear gene *CPLD24* (*Cre10.g435850*) was selected as the internal reference. Total gDNA (genomic DNA) samples from LA-T1, LA-T2, and LA-T3 were extracted and analyzed. Primers targeting *aph8* specifically located on syn-LA and targeting *nad5* located in both syn-LA and OMtG were designed. In a comparison of the amount of *aph8* relative to *nad5* in a tested gDNA, the ratios of syn-LA were 0.236, 0.251, and 0.297 in the three transformants LA-T1, LA-T2, and LA-T3, respectively (Figure 3D). This implies that syn-LA covered about one-third of total mitochondrial DNA copies.

In addition, phenotypes of transformants, considering growth status in dark conditions and the reproductive rate under the concurrent condition, were evaluated to estimate the performance of syn-LA in living cells (Figures 3E,F). The results showed that syn-LA-containing algal cells (LA-T1, LA-T2, and LA-T3) adapted better to dark conditions compared to non-transformed *cc-2654* and *cc-4098*. However, it did not perform as well as wild type cc-124. Reproductive rate analysis showed that LA-T1, LA-T2, and LA-T3 grew faster than *cc-2654* and *cc-4098* but grew slower than cc-124. This result further indicates that syn-LA functions *in vivo* partially rescue the defect growth of *cc-2654* in dark conditions, which was consistent with previous findings that syn-LA only occupied about 30% of total mitochondrial DNA. It was assumed that if syn-LA could replace all OMtG, the defects of *cc-2654* could be fully recovered. Consequently, attempts to increase the syn-LA ratio in transformants were necessary.

### Attempts to increase syn-LA heteroplasmic ratio and gradually lost foreign DNA

**Figure 4:**
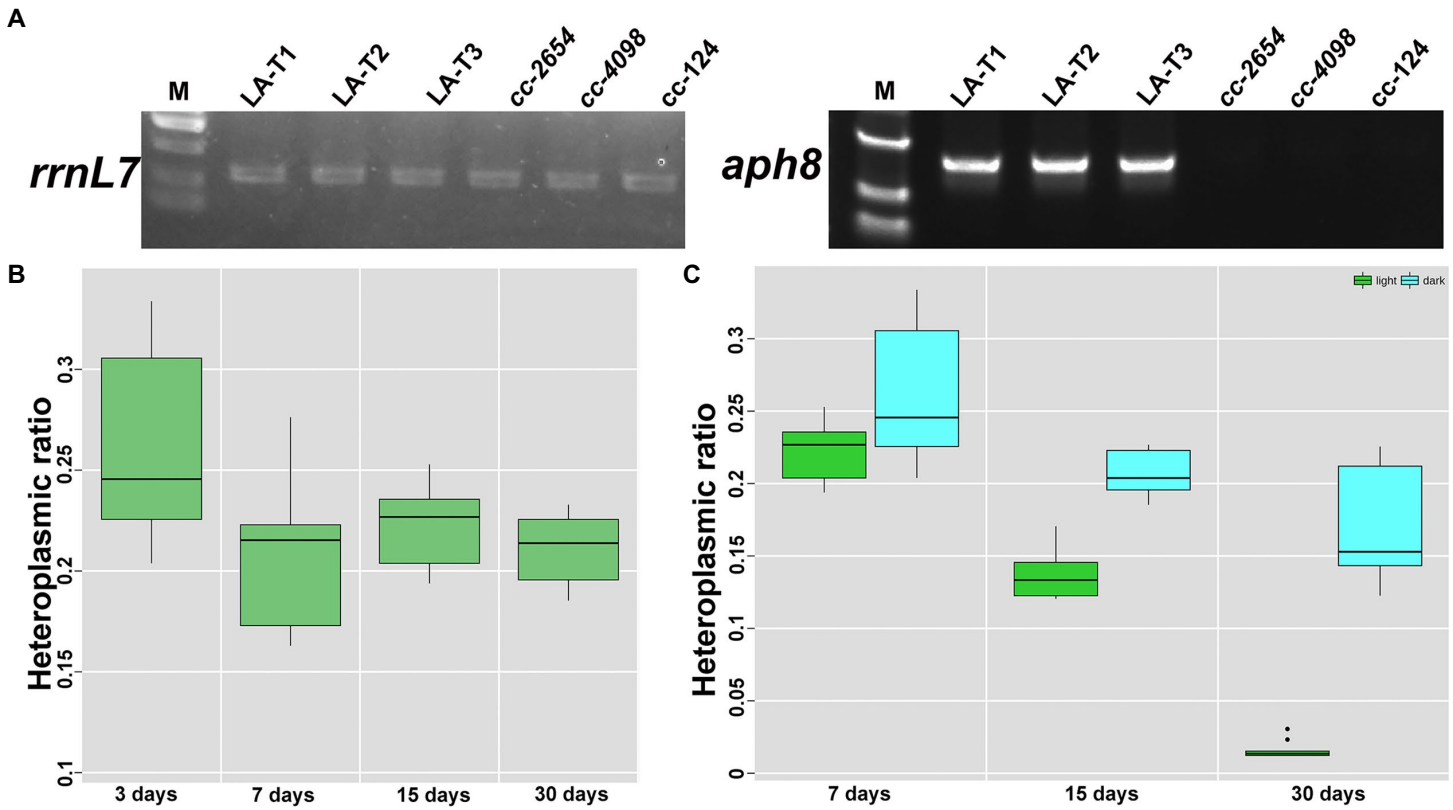
Increase syn-LA heteroplasmic ratio with expressed *aph8* resistance gene. **(A)** RT-PCR results of inserted *aph8* gene taking *rrnL7* as internal control. **(B)** Heteroplasmic ratio of transformants with 10 μg/ml paromomycin stress. **(C)** Heteroplasmic ratio of transformants grown in darkness or in light conditions.

Many studies have suggested that homoplasmic mitochondrial mutations have milder clinical symptoms than heteroplasmic (Finsterer and Mehri, 2022). Thus, the ability to promote the heteroplasmic mutant to homoplasmic would be useful. In this study, resistance gene *aph8* and corresponding paromomycin stress were introduced to increase syn-LA copies in transformants. Certainly, a test to verify *aph8* expression is important before doing this. Thus, reverse transcription PCR amplification was conducted with p1&p4, taking rrnL7 (p5&p6) as internal control (Figure 4A and Supplementary Table 1). Results showed that the *aph8* gene was successfully transcripted. Further, positive transformants were inoculated onto plates containing 0, 8, 10, 12, 14, and 16 μg/ml paromomycin (Supplementary Figure 1C). It was shown that these transformants could grow on plates containing ≤12 μg/ml paromomycin. Surprisingly, long-term antibiotic stress seemed unlikely to increase the syn-LA ratio. Syn-LA heteroplasmic ratios of samples grown on a plate containing 10 μg/ml paromomycin were at the third, seventh, fifteenth, and thirteeth days (Figure 4B). The result showed that no significant increase of syn-LA heteroplasmic ratio can be observed. We hypothesized that mitochondrial genes located in closed compartments might not be as responsive to environmental stress, contrary to what was originally thought.

In addition, we also found that the mitochondria transformants seem to have the ability to exclude transformed DNA. A test to continuously monitor the heteroplasmic ratio showed syn-LA will gradually reduce and disappear within a month in transformants that grow in constant light conditions (Figure 4C), while transformants cultured in darkness had a relative stable syn-LA ratio. This result may be because syn-LA provides the full *cob* function, which is necessary for growth in the dark. This result is not surprising, as mitochondria are reported to normally prevent the entrance of foreign nucleic acids. In natural conditions, apart from very few tRNAs, nucleic acid molecules of other forms, whether DNA or RNA, have difficulty crossing this bound organelle (Mileshina et al., 2011; Shikha et al., 2020; Chandel, 2021).

### Main barriers and optional improvements for the development of artificial mitochondrial genomes

Mitochondrial genome synthesis and intracellular functionalization are undoubtedly meaningful endeavors, whether in the consideration of custom- engineered cell “powerhouses” or treating mitochondrial diseases. However, progress in this field has been slow, and there are barriers that need to be understood. In this study, we assembled a syn-LA of the mitochondrial genome, transferred it into living algal mitochondria, and analyzed the *in vivo* function of the artificial genome. Based on the difficulties we encountered, we propose the following barriers to be considered and discuss optional improvements within this field.

**Figure 5:**
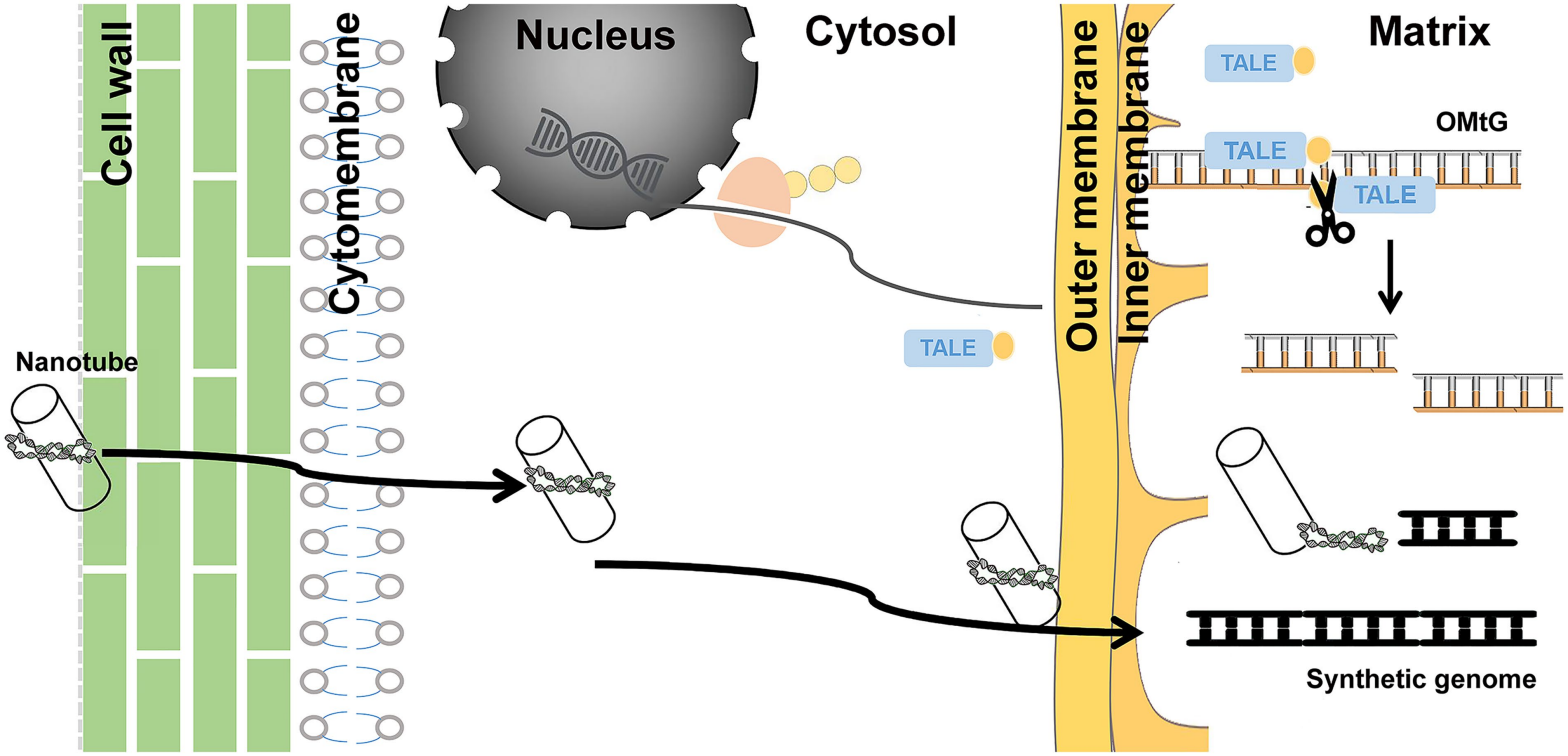
Schematic diagram of methods that could assist in overcoming limitations. Carbon nanotubes used as a carrier to deliver synthesized DNA and mito-TALEN tools steadily expressing cytoplasmic fluid transported into matrix, cutting OMtG, could transform from heteroplasmic to homoplasmic status.

Due to advanced genome sequencing data and established synthesis technologies, the design and assembly of the mitochondrial genome is relatively straightforward in species with simple mitochondrial genome structures. However, the *in vivo* function of synthetic genome could be challenging. As shown in Figure 5, synthetic DNA needs to penetrate the cell wall, cytomembrane, and outer and inner membranes of mitochondria—a total of four layers of physical barriers—to get to the right place to be recognized by the mitochondrial transcription and translation machinery. The natural pores of these barriers may be just a few nanometers in size (Schwab et al., 2015). With conventional DNA delivery systems, it is almost impossible to accomplish such a task. The only biolistic delivery system that is in operation does not exhibit satisfying transformation efficiency. Establishing an efficient delivery system is therefore the first challenge. A recent study showed that custom-coated mitochondria-oriented carbon nanotube transmission systems could be an excellent option (Law et al., 2022). The development of carbon nanotube technology has made sizes of 5–20 nm possible. Through coating with mitochondria-directed peptide and conjugation of foreign DNA, nanotubes can be ideal cargo carriers.

The huge number of mitochondria copies is the second challenge. Foreign DNA entry inevitably leads to coexistence of both. It is difficult to artificially increase the proportion of target genome types to achieve homoplasmic status. Long-term stress selection might be an option, but it did not work well in our study. Recent research on mitochondrion editing suggests that mitochondria-directed transcription activator-like effector nuclease may be a feasible choice (Kazama et al., 2019; Arimura et al., 2020; Forner et al., 2022). A stable hereditary cassette with encoding mito-TALEN tools inserted into the nuclear genome could specifically shear OMtG, regardless of the fact that the synthetic genome may contribute to achieving the relevant homoplasmic status (Figure 5).

Additionally, the lack of screening markers and sorting methods are further limitations. Compared with nuclear transformation screening, efficient screening methods such as resistance screening and fluorescence screening have not been established. The only reliable screening approach with mutant rescue is relatively inefficient and time-consuming. In our study, lengthening the screening period to 3 months was needed. Manual and PCR-based sorting methods further reduced the efficiency and limited the scale. Developing an effective screening and high-flux-sorting system is therefore necessary. One study regarding an intelligent image-activated sorting system based on mitochondrial microscopic morphology differences is interesting in this regard (Harmon et al., 2022). Although there may be other obstacles, the three mentioned above are likely to be the main challenges.

## Conclusion

*Chlamydomonas reinhardtii* is considered to be a sustainable cell factory with substantial industrial prospects for redesigning and reconstructing genomes for relevant purposes. The small and simple mitochondrial genome of this alga makes it ideal for such studies. In this study, we designed and synthesized the left arm of the *C. reinhardtii* mitochondrial genome, successfully transferred it into mitochondria, and verified its function inside *cc-2654* cells. Despite progress, we also encountered some unexpected problems. We then listed the limitations and proposed solutions for these limitations. To summarize, conventional genome synthesis projects typically involve a “design-build-test-upgrade” cycle. This first round study of the mitochondrial genome will undoubtedly provide a valuable reference for future research.

## Materials and methods

### Original mitochondria genome sequence acquisition and design of syn-LA

A 15,758 bp *C. reinhardtii* mitochondria genome of *C. reinhardtii* was obtained from the NCBI database.^1^ All designs for syn-LA including deletions, substitutions, and insertions were carried out using Snapgene 4.1.8.^2^ Insert resistance genes (*aph8*) were codon-optimized based on maximum codon frequency using CodonW _1_4_2 software.

### Strains and growth conditions

The *C. reinhardtii* respiratory deficient strain *cc-2654* (*dum-1,* mt*^−^*) and its alleles *cc-2255* and *cc-4098* were obtained from the Chlamydomonas Resource Center, University of Minnesota, United States. Wild-type strain cc-124 was from our laboratory algae bank. For data collection, all samples were grown in an illumination incubator on Trisacetate-phosphate (TAP) medium cultured at 25°C with shaking at 150 rpm under constant light (40 μmol photons/m^2^/s). All experiments were performed with cells in exponential growth phase. Cells were harvested by centrifugation at 3,000 rpm for 5 min and washed twice with distilled water.

### DNA preparation and PCR amplification analysis

Plasmid DNA was extracted using a Plasmid MiniPrep Kit (Transgen, EM101), and genomic DNA was extracted with a Genomic DNA Kit (Transgen, EE101) following the manufacturer’s instructions. DNA quality was assessed using a NanoDrop2000 Ultra Microscope Photometer (Thermo, United States). PCR amplification was performed with Taq DNA Polymerase (Transgen, AP101) based on a standard three-step program (94°C 3 min; 30 cycles of 94°C 5 s, 60°C 30 s, 72°C 10 s/kb, 72°C 5 min).

### Mitochondria transformation

Transformation was performed as described previously (Hu et al., 2011, 2012) with some modifications. Briefly, exponential growth phase (5–6 × 10^6^ cells ml^−1^) algae cells were collected by centrifugation and spread on the center of a plate containing TAP medium and cultured overnight. A mixture of 0.6 μm gold and 50 nm tungsten particles (10 + 10 mg) was precipitated in a 1.5 ml Eppendorf tube. The pellet was washed with 70% ethanol and sterile water for 20 min, respectively, with strong oscillation. The mixture was collected by centrifuging and stored in 500 μl 50% glycerol. Before bombardment, 50 μl of the stored mixture of particle carriers, 5 μg DNA, 50 μl 2.5 M CaCl_2_, and 20 μl spermidine were added to a 1.5 ml tube, vortexed, and centrifuged. The pellet was washed with 70% ethanol twice, and 200–250 μl pure ethanol was added and vortexed. A 20 μl volume of the mixture was placed on a membrane carrier, air-dried for 10 min, loaded into the biolistic apparatus (Bio-Rad, Hercules, CA, United States), and bombarded at 1,550 psi with helium. Cells were washed and maintained on the TAP medium in darkness for 24 h, then spread on a TAP plate and incubated in darkness for 3 months until green cells had disappeared completely. Subsequently, colonies could be seen after about 7 days of light restoration.

### Mitochondrial protein extraction and western blotting

Five liters of exponential growth phase algal cells were harvested by centrifugation at 5,000 g for 5 min, washed twice with distilled water, and resuspended in 5–10 ml buffer A [0.6 M mannitol, 10 mM Tris–HCl, 5 mM EDTA, 0.2% w/v (0.2 g/100 ml) Bovine serum albumin (BSA), and 0.3% w/v polyvinylpyrrolidone 40 (pH = 7.8)]. Intracellular constituents were released by sonication on ice at 240 W for 15 min with 5 s pulses and 5 s pauses. Low-speed centrifugation at 1,000 g and 4°C was performed twice to remove large debris. The supernatant was centrifuged at 15,000 *g* and 4°C, yielding raw mitochondria.

Proteins of mitochondria were then released in lysis buffer [8 mM Urea, 1% SDS, 40 mM Tris-base, pH = 8.5, 10 mM Dithiothreitol (DTT), and 2 mM Ethylene Diamine Tetraacetic Acid (EDTA) by sonication at 300 W for 20 min with 10 s pulses and 10 s pauses]. Lysates were centrifuged at 15,000 *g* for 10 min at 4°C, and the supernatant was collected and quantified with a Protein Quantitative Kit (Transgen, DQ111). A 20 μg sample of protein was mixed with SDS-PAGE loading buffer, boiled for 5 min, separated by 4–12% SDS-PAGE, and transferred onto a PVDF (polyvinylidene difluoride) membrane (0.2 μm; Millipore, Massachusetts, United States). Membranes were incubated with blocking buffer for 2 h then incubated with primary antibodies for 9 h at 4°C. Membranes were then incubated with secondary antibody for 2 h at room temperature after washing with TBST three times for 10 min each. Finally, membranes were incubated in enhanced chemiluminescence (ECL) substrate buffer at room temperature for 3 min and visualized by a Tanon-5200 Chemiluminescent Imaging system (Tanon Science and Technology, China).

### Analysis of mitochondrial heteroplasmic ratio

The mitochondrial heteroplasmy ratio is a statistic-based real-time PCR, according to published research (Machado et al., 2015). Briefly, genomic DNA was extracted with a Genomic DNA Kit (Transgen, EE101). A NanoDrop2000 Ultra Microscope Photometer device was used to assess sample quality and set a unified concentration of 50 ng/μl. The *CPLD24* (*Cre10.g435850*) nuclear gene with only a single copy (Predict online: https://busco.ezlab.org/) was selected as a reference gene, and H01&H02 primers were designed. H03&H04 and H05&H06 primer pairs were used to amplify *aph8* and *nad5*. Real-time PCR with the DNA samples prepared above was performed using a qPCR SYBR Green Kit (Yeasen, 11143ES50) on an ABI QuantStudio 6 Flex Detection Device as recommended by the manual. Both *aph8* and *nad5* were normalized against *CPLD24* according to the 2^−ΔΔCt^ method. Calculating the relative amount _(*aph8*)_/relative amount _(*nad5*)_ gave the heteroplasmy ratio of syn-LA as *aph8* specifically located on syn-LA, while *nad5* is located on both.

### Statistical analysis

For statistical analysis, *t*-tests were conducted (^*^0.01 < *p* < 0.05, ^**^0.001 < *p* < 0.01, and ^***^p < 0.001). Results are mean values ± SD.

## Data availability statement

The datasets presented in this study can be found in online repositories. The names of the repository/repositories and accession number(s) can be found in the article/Supplementary material.

## Author contributions

ZH, GZ, and QW designed the research. QW, HL, JZ, GZ, and XL performed experiments and data analyses. QW wrote the manuscript. DH helped with revising and refinement of language in the manuscript. All authors contributed to the article and approved the submitted version.

## Funding

This research was funded by National Natural Science Foundation of China (32273118 and 41876188), Chinese National Key R & D Project for Synthetic Biology (2018YFA0902500), Shenzhen Basic Research Projects (JCYJ20180507182405562), and Shenzhen Special Fund for Sustainable Development (KCXFZ20211020164013021).

## Conflict of interest

The authors declare that the research was conducted in the absence of any commercial or financial relationships that could be construed as a potential conflict of interest.

## Publisher’s note

All claims expressed in this article are solely those of the authors and do not necessarily represent those of their affiliated organizations, or those of the publisher, the editors and the reviewers. Any product that may be evaluated in this article, or claim that may be made by its manufacturer, is not guaranteed or endorsed by the publisher.

## Supplementary material

The Supplementary material for this article can be found online at: https://www.frontiersin.org/articles/10.3389/fmicb.2022.1064497/full#supplementary-material

## Footnotes

^1^ https://www.ncbi.nlm.nih.gov/nucleotide/NC_001638.1

^2^ https://www.snapgene.com/
