# Supplementary material for: Chemical synthesis of left arm of Chlamydomonas reinhardtii mitochondrial genome and in vivo functional analysis
Source: Front Microbiol. 2022 Dec 22;13:1064497. doi: 10.3389/fmicb.2022.1064497 (PMC9813849; doi:10.3389/fmicb.2022.1064497)
Supplement: Supplementary file 2 [file Table_2.DOCX]

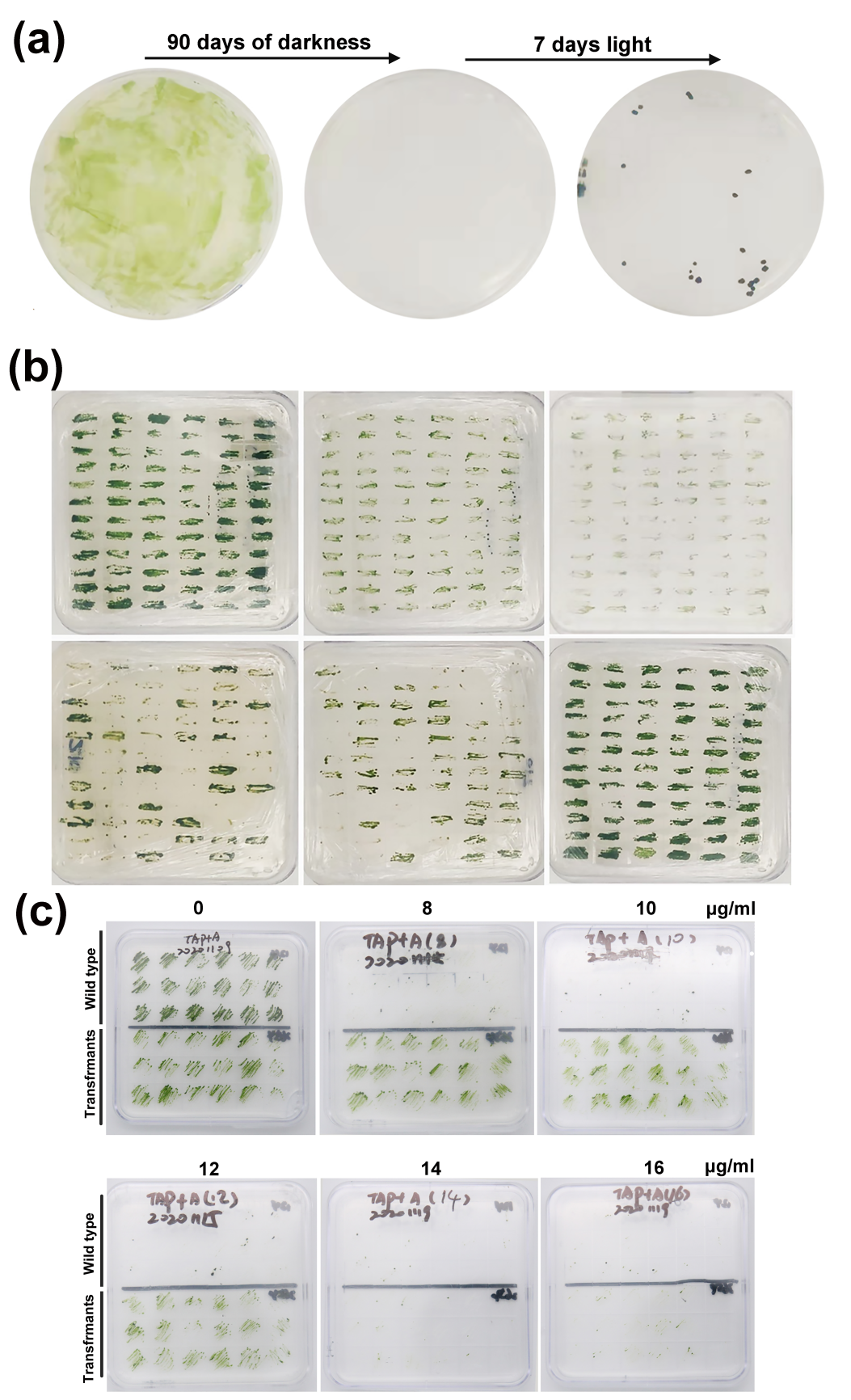


**Supplementary Figure 1.** **(a)** Image showing process of screening mitochondrial transformats; **(b)** transformats obtained from multiple several transformation events.**(c)** Growth status of transformants cultured on TAP plate contains 0, 8, 10, 12, 14 and 16 μg/ml paromomycin.
